# A qualitative study on HIV positive women experience in PMTCT program in Indonesia

**DOI:** 10.1186/1742-4690-9-S1-P119

**Published:** 2012-05-25

**Authors:** Martiani Oktavia, Anita Alban, Prisca AC Zwanikken

**Affiliations:** 1 IMPACT Program with Padjadjaran University and Hasan Sadikin Hospital Bandung, Indonesia; 2 University of Copenhagen, Denmark; 3 Royal Tropical Institute, The Netherlands

## Introduction

Indonesia has one of the fastest growing HIV epidemics in South-East Asia, which was largely driven by injecting drug users (IDUs). A projection model suggests that there will be a shifted of HIV epidemic from contaminated needles among IDUs to their sexual partners via heterosexual contact. At present, women are accounted 25% of all reported AIDS cases cumulatively. Despite the growing need for prevention mother to child transmission (PMTCT) of HIV is emerging, coverage of intervention for HIV test and ARV prophylaxis among HIV pregnant women are still low. This study calls for more client-oriented PMTCT program based on women's need and demand in a changing HIV epidemic.

## Material and method

Mixed study design, which consists of a literature review and a qualitative study with in-depth interview among HIV positive women with history of PMTCT (purposive sampling).

## Results

Nine women aged 25 to 33 years old were selected as respondents for qualitative study. Majority of them had contracted HIV from their spouse, who formerly injected drugs. All of them perceived low or no risk factor for HIV. HIV positive women valued high acceptance of HIV testing in primary health care with conditions; ensure confidentiality and quality of counselling. Lack of information about PMTCT and unintended pregnancies presumably correlated with late initiation of ARV prophylaxis among HIV infected pregnant women. Despite almost all of the respondents were expressing no intention to have more children, there was unmet need for contraception. Stigma and discrimination remain exist in various forms; fear of being isolated/separated from friends and family, sub-optimal treatment in hospitals by healthcare workers.

## Conclusions

Findings from this study provide a basis for establishing PMTCT program responsive to the need and demand of women as subject of intervention. Comprehensive interventions need to be integrated into existing health systems and utilize resources at the locals disposal. A successful and sustainable PMTCT of HIV program requires a close collaboration with stakeholders e.g. governmental institutions, non-governmental organisations, and civil society representatives.

